# *BJPsych Open*: a summary of growth and vision for my second innings as editor-in-chief

**DOI:** 10.1192/bjo.2023.35

**Published:** 2023-03-23

**Authors:** Kenneth R. Kaufman

**Affiliations:** Departments of Psychiatry, Neurology and Anesthesiology, Rutgers Robert Wood Johnson Medical School, New Brunswick, New Jersey, USA; Department of Psychological Medicine, School of Academic Psychiatry, Institute of Psychiatry, Psychology & Neuroscience, King's College London, UK

**Keywords:** *BJPsych Open*, editor-in-chief, remit, editorial board, growth, productivity and quality, metrics, thematic series, vision, ethics

## Abstract

As my second 5-year term as its editor-in-chief begins, it is important to review what *BJPsych Open* has accomplished, its areas of growth and what should be our future vision for the Journal. The keyword throughout this editorial is growth, with emphasis on growth in quality, for meaningful growth can only exist with increased quality. The original remit remains the correct long-term direction for the Journal, with the important modifier ‘relevance’ added to ensure quality – a general psychiatric journal with high-quality, methodologically rigorous and relevant publications, with relevance to the advancement of clinical care, patient outcomes, the scientific literature, research and policy. During this second term, I desire to expand the editorial board to fill expertise and diversity gaps; increase editorials and commentaries highlighting specific articles and timely events with psychiatric themes; focus on thematic series driven by the editorial board; and address under-represented topics.

Each editor-in-chief (EIC) has a vision when assuming the role. It is hard to realise that 5 years have now passed as I enter my second term as EIC for *BJPsych Open*. What was my vision then and what have we accomplished? To maintain our current recognition in the scientific community, to have continued growth and to ensure high-quality submissions and publications all require significant and integrated efforts by the editorial and publication teams. To have a meaningful vision statement, it is important to understand where one has come from to contextualise where one wants to go.

And those enlightened readers who follow the game played with a wooden bat and a red leather ball know that in test cricket the second innings is decisive. Here, as EIC of *BJPsych Open* (the team captain, as it were), I am in the privileged position of once again leading a splendid team onto the field that has had a good first innings (my first term as EIC), and so it is fitting that we now plan for an even more successful second term effort, recognising what ‘worked in the first innings’ and what should be ‘maintained/strengthened/added in the second innings’.

Let us view these issues historically while addressing my plans for the future of *BJPsych Open* and ‘the second innings.’

## Journal creation and original remit

When *BJPsych Open* was conceived in 2014 (the first issue was published in June 2015), the Royal College of Psychiatrists (RCPsych) and the Editor of the *British Journal of Psychiatry* (*BJPsych*) recognised a key failing of print publishing – the lack of available print space to publish good-quality articles with appropriate scientific rigour. In essence, the *BJPsych* could focus on publishing only ‘the best of the best’. Yet what happened to those other ‘good articles’, papers that deserved to be published? Would College members have the opportunity to read – and to publish – these in a College journal? A key mission of the College is dissemination of knowledge to its members, the scientific community and the public – and the inability to publish methodologically rigorous articles ran counter to this mission. The College and the College Editor (Professor Kamaldeep Bhui) had foresight – their solution was the creation of *BJPsych Open*, which matched the changing landscape of academic publishing and the creation of Plan S.^1–5^ The open access format of *BJPsych Open* has permitted significant growth since the Journal's inception and especially during my first 5-year term as EIC.

## Editorial board and growth

The fundamental element of all successful journals is a functioning editorial board. I have endeavoured to create a board based on meritocracy that is both gender neutral and representative of diverse backgrounds. Board membership is dynamic, as all board members have other obligations affecting their ability to serve on the board. I appreciate this and have focused on making the board experience collegial and enjoyable – by so doing, and by keeping an open door to all board members, the turnover of board members is minimised, and often all that is required is a brief hiatus in handling papers. To maximise editorial efficiency, along with the managing editor Anna Munks, I routinely review the productivity of handling editors and transition them accordingly. I have virtual or telephone communications with board members between board meetings, for I treasure the board, and by so doing have created an ongoing positive experience that translates into a more successful journal.

In 2015, the founding editorial board was comprised of only seven members (five men and two women); the leadership included Professor Kamaldeep Bhui, EIC (UK), Professor Gin Malhi, Deputy Editor (Australia) and myself, Deputy Editor (USA). As noted in a previous editorial, the editorial board grew in both numbers and diversity and by the end of 2019 (2 years into my first term as EIC), the board had been expanded to 38 (17 men and 21 women) from 15 countries.^4^ Now, at the conclusion of my first 5-year term, I have further increased the board to 72 members (37 men and 35 women) from 20 countries, not including a recently added statistical panel. Further expansion will naturally occur to fill current subject expertise gaps and as required to address thematic series.

## Journal productivity and growth

*BJPsych Open* was conceived as a cascade journal^2–4^ – authors of methodologically sound and meaningful articles that were not accepted by *BJPsych*, often because of rigid print space limitations, were offered the opportunity for these articles to be transferred, reviewed and published in *BJPsych Open*, the new sister journal in the RCPsych–CUP portfolio (Royal College of Psychiatrists – Cambridge University Press). Now most submissions to *BJPsych Open* are independent submissions as opposed to transfer submissions; furthermore, *BJPsych Open* itself transfers submissions to other portfolio journals as indicated. Although *BJPsych Open* was not initially indexed, now it is indexed in Current Contents, Web of Science, PsycInfo, Scopus, PubMed, Embase, ProQuest and DOAJ. Further, as an open access journal, *BJPsych Open* has been compliant with Plan S from the start.

The Journal has continued to grow since its inception. Specific productivity metrics for each volume (calendar year) since I have been EIC (Table 1) show sustained growth in publications, with papers covering multiple areas in general psychiatry. The diversity of submissions is reflected in their corresponding authors, who represent 70 countries.

**Table 1:** *BJPsych Open* growth in productivity

Volume	Year	Papers	Pages
4	2018	76	518
5	2019	95	683
6	2020	138	970
7	2021	208	1487
8	2022	199	1572

## Quality, impact factor and growth

An increased number of papers published may denote journal growth but does not necessarily indicate improved quality. Several proxies may be considered: more prestigious indexing (which has occurred); growth in total annual citations (to 2654 in 2022); total citations during my five-year first term (7101 citations in 2018–2022); growth in the number of full-text articles downloaded (to >679 000 in 2022); growth in mentions as tracked by Altmetric (to >4300 in 2021); the Journal's *h*-index (at time of submission, the CrossRef *h*-index was 38); the Journal's CiteScore (at time of submission, the CiteScore was 4.3). Perhaps the most widely used, but flawed, metric is the impact factor – for the impact factor can be skewed by highly cited individual articles: compare COVID-19 pandemic articles, which may be highly cited over a brief time period,^6^ with research articles and reviews, which may have delayed but extended citations over decades.^7^ Indeed, at present the most highly cited paper in *BJPsych Open*, with 290 citations, is the COVID-19 article ‘Anxiety, depression, traumatic stress and COVID-19-related anxiety in the UK general population during the COVID-19 pandemic'^6^ (the most cited papers in *BJPsych Open* are listed at www.cambridge.org/core/journals/bjpsych-open/most-cited). Although when I took over as EIC in the autumn of 2017 I estimated ‘achieving a target impact factor of 2–3 within 5–7 years’,^3^ *BJPsych Open* has witnessed a significant increase in its impact factor over the course of just 3 years: from 2.286 in 2019 (its inaugural impact factor) to 3.209 in 2020 and 5.165 in 2021. Table 2 summarises the growth in specific metrics for *BJPsych Open* (citations, full-text downloads and impact factor) during my first term as EIC (2018–2022) and a wider range of metrics for 2021 are given on the Journal's website (www.cambridge.org/core/journals/bjpsych-open/information/about-this-journal/journal-metrics).

**Table 2:** *BJPsych Open* growth in impact factor, citations and full-text downloads

Year	Impact factor^a^	Citations^a^	Full-text downloads^b^
2018	n.a.	364	46 043
2019	2.286	716	98 939
2020	3.209	1144	193 134
2021	5.165	2223	619 681
2022	n.a.	2654	679 120
Total		7101	1 636 917

n.a., not applicable.

a. Data from Journal Citation Reports^TM^.

b. Downloads recorded by Cambridge University Press.

Further, the 5-year impact factor has correspondingly increased to 4.66. It is noteworthy that the Journal’s impact factor grew at the same time as we saw growth in the number of articles published and the median number of citations per article, all of which are suggestive of increased quality across the Journal. Quality can be enhanced by greater editorial scrutiny regarding research integrity and publication ethics – these are constantly being addressed by the editorial team and the RCPsych portfolio is publishing a series of editorials addressing these issues.^8–11^ Quality can also be enhanced by paying greater attention to statistics and methodology – *BJPsych Open* now has a statistical panel including methodologists. Finally, quality can be enhanced by focusing on the relevance of specific submissions to meaningfully advance mental health. The true quality of *BJPsych Open* will not be measured by metrics but rather by the effect it has on the scientific literature, research, policy, clinical care and patient outcomes, which will only be determined over time.

## Thematic series and growth

As an open access journal with freedom from print limitations, thematic series have been created under my tenure. Topics for thematic series are recommended both by the EIC and editorial board members and, as indicated, are interdisciplinary, with consideration of cultural and ethical issues. Thematic series are a collection of research articles, reviews, short reports, editorials and commentaries that address specific topics in detail and are created through targeted submissions selected by series lead editors and by an open call for submissions. Articles published after the release of the thematic series can be added to the collection, enhancing the experience of readers and serving to constantly update the literature. New features for these thematic series include the addition of digital content (podcasts and videos) (www.cambridge.org/core/journals/bjpsych-open/videos-and-podcasts) and a link to all collections (www.cambridge.org/core/journals/bjpsych-open/collections).

Our published thematic series (and their editorial leads) are:

- Emerald Series (https://www.cambridge.org/core/journals/bjpsych-open/emerald-series) (Graham Thornicroft)^12^ – released 2019
- Cognition in Mood Disorders (https://www.cambridge.org/core/journals/bjpsych-open/themed-series-cognition-in-mood-disorders) (Allan Young, Richard Porter, Katie Douglas)^13^ – released 2020
- COVID-19, Healthcare and Healthcarers (https://www.cambridge.org/core/journals/bjpsych-open/bjpsych-open-covid-19-healthcare-and-healthcarers-themed-series) (Richard Williams, Kenneth Kaufman, Esther Murray)^14^ – released 2022
- Refugee and Asylum Mental Health (https://www.cambridge.org/core/journals/bjpsych-open/bjpsych-open-refugee-and-asylum-mental-health-themed-series) (Cornelius Katona, Derrick Silove, Kenneth Kaufman, Pieter Ventevogel)^15^ – released 2022.

Thematic series in progress (partially published) with editorial leads include:

- Biomarkers of Dissociation (Dick Veltman, Simone Reinders, Allan Young)^16^
- Emergencies, Major Incidents, Terrorist Attacks and Mental Health (Richard Williams, Caroline Bell)^17^
- Neuropsychiatry (Marco Mula, Niruj Agrawal, Jay Salpekar).^18^

Planned thematic series with editorial leads include:

- Human Rights Based Mental Healthcare: New Developments (Helen Herrman, Silvana Galderisi, Paul Appelbaum, Norman Sartorius)
- Non-suicidal Self-Harm (Ted Petti, Kathryn Cullen)
- Immune Dysfunction in Mental Disorders (Ishrat Husain, Rebecca Strawbridge, Allan Young)
- Digital Mental Health (John Torous)
- Mental Healthcare Economics (Judit Simon).

## Vision for the second term

Growth is the keyword in this editorial's subheadings. A question posed is whether the original *BJPsych Open* remit should change with its growth and international recognition. I believe the original remit is the correct long-term direction for the Journal – a general psychiatric journal with high-quality, methodologically rigorous publications. Consider the vision themes summarised in my first editorial as EIC, which remain relative constants:^3^

- no high-quality paper that is methodologically sound, original and with clinical relevance is rejected
- all specialties, subspecialties and associated fields have space
- continues to publish a wide range of article types (editorials, research articles, reviews, short papers, exceptional case reports, case series, commentaries, protocols and special communications)
- maintains a strong editorial board with an international base
- publishes at least one thematic series annually
- remains an egalitarian journal, economically accessible for all authors who desire to publish
- continues to grow in submissions, authors, acceptances and reputation
- is both author friendly and highly readable.

On careful reflection with especial consideration of increasing submissions during my tenure as EIC, a qualifier needs to be added to our remit to ensure increased quality and meaning of future publications, ‘relevance’. Thus, our remit now becomes ‘a general psychiatric journal with high-quality, methodologically rigorous and relevant publications, with relevance to the advancement of clinical care, patient outcomes, the scientific literature, research and policy’.

There is no target impact factor for this journal – rather, an impact factor sufficient to ensure quality submissions and continued international recognition of the Journal. The emphasis in growth will be in growth of quality, for meaningful growth of the Journal can only exist in the context of increased quality. With growth there will be changes – editorial board expansion and restructuring to best handle submissions with maximal editorial board and reviewer expertise; increased editorial board and editorial office screening, to ensure that submissions adhere to journal and RCPsych portfolio policies; changes to match changes in the publishing landscape as open access and Plan S evolve.

*BJPsych Open* is a society journal dedicated to the maximal dissemination of knowledge and will continue to be responsive to its ‘audience (clinicians, clinical academics, researchers and policy makers) as well as authors, reviewers and editorial board members concerning what should be done to further improve quality and breadth of the Journal’.^4^

During my second term, the Journal will focus on increased numbers of editorials and commentaries to highlight the significance of specific articles as well as research, clinical and policy themes and to specifically address timely important events with psychiatric themes. *BJPsych Open* published two of the first editorials addressing the global mental health response to the COVID-19 pandemic^19^ and mental health responses required for Ukrainian refugees.^15^ There will be continued focus on editorial board-driven thematic series, including interdisciplinary topics and authorship. There will be increased use of digital content for both thematic series and specific articles. Further, under-represented topics will be emphasised: advocacy, mental health in low- and middle-income countries, ethnic minorities, refugees, implementation, ethics, human rights, sport psychiatry, and arts and mental health.

My vision as EIC of *BJPsych Open* would not be possible without our authors, dedicated reviewers, editorial board members, and editorial and publishing offices. I am privileged and honoured to serve as EIC of this journal and remain thankful for the efforts of so many in making *BJPsych Open* a viable and increasingly important journal in the scientific literature.

## Data Availability

Data availability is not applicable to this editorial as no new data were created or analysed in its preparation.
